# Regenerative and Protective Effects on Dental Tissues of a Fluoride–Silicon-Rich Toothpaste Associated with a Calcium Booster: An *In Vitro* Study

**DOI:** 10.3390/dj11060153

**Published:** 2023-06-14

**Authors:** Fabiano Vieira Vilhena, Simone dos Santos Grecco, Alejandra Hortencia Miranda González, Paulo Henrique Perlatti D’Alpino

**Affiliations:** 1 Trials Research and Development, Rua Maria Benedita de Oliveira, 1-25, Bauru 17018-776, Brazil; fabianovilhena@yahoo.com.br; 2 Triplet Biotechnology Solutions, Av. Nossa Senhora de Fatima, 11-31, Bauru 17017-337, Brazil; grecco.simone@gmail.com (S.d.S.G.); alejandra.horten@gmail.com (A.H.M.G.); 3 School of Sciences, São Paulo State University (UNESP), Av. Eng. Luís Edmundo Carrijo Coube, 14-01, Bauru 17033-360, Brazil

**Keywords:** dentin, tooth remineralization, toothpaste, booster, microscopy, electron, scanning

## Abstract

Calcium boosters have been used as a supplement for fluoride toothpastes to repair the dental tissues and reduce dentin permeability. This in vitro study aimed to characterize the regenerative and protective effects of the treatment of dental tissues with a fluoride–silicon-rich toothpaste associated with a calcium booster. Bovine enamel and dentin blocks (*n* = 5) were obtained (4 × 4 × 6 mm). A fluoride–silicon-rich toothpaste and a calcium booster were used to brush the enamel and dentin both immediately and five days afterwards. The outcomes were then compared to those of the untreated control group. After that, the specimens were cross-sectioned. SEM was used to evaluate the micromorphology of the surface and cross-section. Energy-dispersive X-ray spectroscopy (EDS) was used to determine the elemental analyses (weight%). After treatment for 5 days with a booster/silicon-rich toothpaste, EDS analysis demonstrated that it induced a significant mineral change. It was also able to form a protective silicon-enriched mineral layer on both enamel and dentin surfaces. It was demonstrated in vitro that a fluoride–silicon-rich toothpaste associated with a calcium booster regenerates the dental tissues, remineralizing the enamel structure and occluding the dentin tubules.

## 1. Introduction

Dental erosion is considered a disorder of modern life mainly due to changes in nutritional habits and lifestyle [1]. Fluoride toothpastes are the most affordable oral hygiene products for the population to prevent and treat dental erosion [2]. In light of the prevalence of tooth erosion, researchers and oral care companies are working to develop new toothpastes that will maintain and enhance the clinical efficacy of fluoride [3,4]. Different substitute ion concentrations, either absorbed into the apatite lattice or adsorbed onto the crystal surface, can improve the remineralization process [5,6]. Remineralization is a natural tooth repair process that involves the “redeposition” of minerals lost by enamel and/or dentin [7]. Each ionic grouping of the hydroxyapatite molecule can be replaced by another ionic group of the same or different valence due to the high flexibility of its structure [8].

Given this flexibility, the development of new products is concentrating on the ionic substitution at the various hydroxyapatite sites (calcium, phosphate, and hydroxyl sites) [8]. For example, fluorine ions (F-) can take the place of hydroxyl ions (OH-), and this substitution tends to make hydroxyapatite less soluble [9]. It has been observed that adding silicon, or silicate, to the remineralizing compound hydroxyapatite increases its bioactivity and ability to generate apatites [10,11]. Other active ingredients have been incorporated as desensitizing agents in the formulation, such as calcium carbonate, arginine, and strontium acetate, in order to protect the dental tissues against erosive attacks, but their effects on dentin are still limited [12].

In spite of the alleged efficacy of fluoride toothpastes to control tooth structure loss against erosion [13], recent reports have raised concerns over the increased prevalence of erosive wear [14]. A recent systematic review concluded that the evidence of the protective potential of fluoride toothpastes in erosive and abrasive models was, for many reasons, graded as moderate for conventional toothpastes and low for the association of stannous with fluorides [14]. In dentin, most of these agents aim to occlude the dentinal tubules, but previous studies demonstrated that these ingredients act as desensitizing agents to reduce dentin hypersensitivity [15]. Clinical studies today provided no clear evidence of the protective agents available in fluoride toothpastes against dental erosion [14].

In the scientific and technological area, dental regeneration was initiated with the launch of “fluoride booster” formulations [16]. According to a recent systematic analysis, the evidence of fluoride toothpaste’s preventive potential in erosive and abrasive models was graded as moderate for conventional toothpastes and low for the interaction of stannous with fluorides for a variety of reasons [14]. Because these new products contain bioactives based on hydroxyapatite and calcium phosphates [17], boosters were added to toothpaste formulations in this way to increase fluoride effectiveness and accelerate remineralization, the process of incorporating calcium and phosphate into the demineralized tooth structure that leads to mineral gain [18].

Boosters are known to increase fluoride effectiveness and accelerate remineralization, the process of incorporating calcium and phosphate into the demineralized tooth structure, which results in mineral gain [18] because they contain bioactives based on hydroxyapatite and calcium phosphates [17]. Occluding the exposed dentin tubules and lowering dentin permeability appear to be more effective and consistent treatments for dentin hypersensitivity [19]. Fluoride boosters, which are biomimetic substances included in toothpaste formulations, are used for this purpose [20]. These substances serve as carriers for calcium and fluoride inside dental tissues, which increases their efficacy as remineralizing agents by strengthening tooth structure by enlarging hydroxyapatite crystals, which become less soluble and porous [21]. Boosters may enhance the destructive mineral layer’s precipitation onto the dentinal tubule apertures and/or inside the tubules, reducing the dentin sensitivity brought on by this fluid movement [22].

The goal of the present in vitro study was to characterize biomimetic/bionic action of a calcium booster associated with a fluoride–silicon-rich toothpaste on the regeneration and protective effect on dental tissues to prevent mineral loss.

## 2. Materials and Methods

### 2.1. Products Tested

The characteristics of the products are displayed in Table 1.

### 2.2. Specimen Preparation of Dental Enamel

Bovine incisors that were recently extracted and in excellent condition were chosen, cleaned, and kept in a 0.1% thymol solution at 4 °C until needed. Using a diamond-impregnated disc (Extec, Enfield, CT, USA) and a cutting machine (IsoMet 1000, Buehler, Lake Bluff, IL, USA), enamel blocks (4.0 × 4.0 mm) were cut from bovine incisors [11]. After that, epoxy resin (EpoxiCure Epoxy Resin and Hardener, Buehler, Lake Bluff, IL, USA) was used to embed the enamel blocks (*n* = 5). The surfaces were subsequently wet-polished using a polishing machine (Single Grinder Polisher, Buehler, Lake Bluff, IL, USA) with 600-grit SiC paper at low speed and 1200-grit SiC paper at high speed. Wet felt wheels and 1 m diamond paste were used for the final polishing (Buehler, Lake Bluff, IL, USA).

Using an electric toothbrush (Oral-B Vitality Precision Clean rechargeable toothbrush), the enamel blocks were first brushed for five minutes (120 cycles per minute, 22 °C) with equal quantities of the booster and fluoride toothpaste (0.3 g each). The toothbrush heads were attached in a structure that made it possible for them to be parallel to the surfaces of the specimens [23]. With an axial load of 200 g, the specimens were brushed linearly [24]. The toothbrush heads were replaced for each application. The blocks were then exposed daily for 3 min for 5 days to equal portions of fluoride and booster toothpaste. The specimens were maintained in solution after the enamel surfaces were treated for additional study.

### 2.3. Specimen Preparation of Dental Dentin

The same procedure used to produce dentin blocks (*n* = 5) was used. The surfaces of the blocks were wet-polished using 600-grit SiC paper at low speed and 1200-grit SiC paper at high speed using a polishing machine. The blocks were also implanted in epoxy resin. Dentin blocks were initially brushed with equal portions of the booster and fluoride toothpaste (0.3 g each) using an electric toothbrush (Oral-B Vitality Precision Clean rechargeable toothbrush) for 5 min (120 cycles per minute, 22 °C). The specimens were then rinsed with deionized water for 5 s and stored in physiological solution. The blocks were then exposed daily to equal portions of booster and fluoride toothpaste for 3 min for 5 consecutive days. After treating the dentin surfaces, the specimens were also stored in physiological solution for further analysis.

### 2.4. Characterization of the Enamel and Dentin Surfaces and Cross-Sections via Scanning Electron Microscopy (SEM) Imaging Observation and Energy-Dispersive X-ray Spectroscopy (EDS)

The morphological analysis of the specimens was performed using a scanning electron microscope (15 kV, TESCAN VEGA3, LMU, Kohoutovice, the Czech Republic). The blocks that were previously sputter-coated with gold in a vacuum evaporator (MED 010; Balzers, Balzers, Liechtenstein). The specimens were then microscopically analyzed, and photomicrographs of the superficial morphology were obtained. To determine a qualitative elemental analysis of the specimens, an EDS point analysis (80 mm^2^, SDD (silicon drift detector), Oxford Instruments, Concord, MA, USA) was performed (high vacuum mode, accelerating voltage 15 Kv). The means were computed for five randomly chosen points (300 µm^2^ each).

The specimens were longitudinally sectioned under water cooling to obtain cross-sections for the subsurface analysis. The cross sections were analyzed using SEM combined with EDS after being dehydrated in silica gel for three hours.

## 3. Results

**Figure 1:**
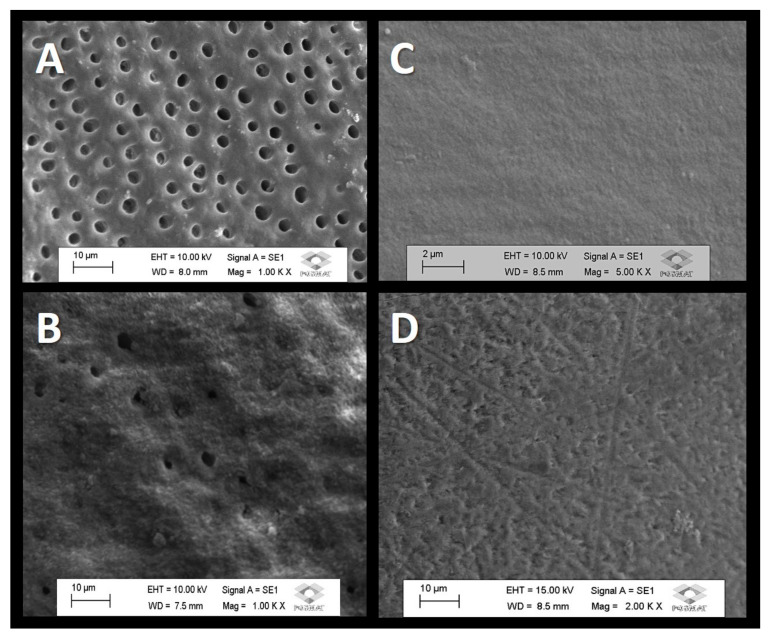
Representative scanning electron micrographs of the morphological analysis of the surface areas: (**A**) demineralized dentin (control) with open tubules; (**B**) enamel surface (control); (**C**) mineralized layer was formed above the dentin immediately after first brushing with toothpaste associated with calcium booster. (**D**) A mineralized layer was formed above the enamel immediately after first brushing with fluoride toothpaste associated with a calcium booster. EHT: electron high tension. Mag: magnification. WD: working distance.

Figure 1 shows the scanning electron micrographs of the morphological analysis of the superficial areas of the dentin and enamel before and after the immediate treatment with the association of a fluoride toothpaste with calcium-booster technology. A scanning electron micrograph of the exposed dentin tubules in the superficial region is displayed in Figure 1A. Following the use of toothpaste, a mineralized layer with partially open dentin tubules can be observed in Figure 1B. A scanning electron micrograph of the control enamel’s surface is depicted in Figure 1C. In Figure 1D, a micrograph of the enamel demonstrates a mineralized area after the immediate treatment with the association of a fluoride–silicon-rich toothpaste with calcium-booster technology.

**Figure 2:**
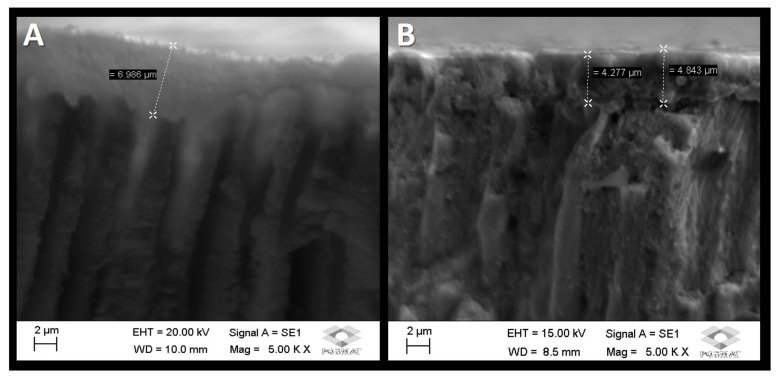
Representative scanning electron micrographs of the morphological analysis of the subsurface areas: (**A**) A mineralized layer was formed above the dentin after five applications of fluoride toothpaste associated with a calcium booster. (**B**) mineralized layer was formed above the enamel after five applications of fluoride toothpaste associated with calcium booster. EHT: electron high tension. Mag: magnification. WD: working distance.

Figure 2 displays scanning electron micrographs of the morphological analysis of the cross-sectional areas of the dentin and enamel. In Figure 2A, a mineralized layer was formed on the dentin surface after five daily applications of the fluoride–silicon-rich toothpaste associated with the calcium booster. The mineralized layer that formed was around 7 µm. It was also demonstrated that there are mineralized areas inside the dentin tubules, which are characterized by whiter morphological areas that partially fill some of these structures. In Figure 2B, it can also be observed that a mineralized layer forms on the enamel after a 5-day daily application of fluoride toothpaste associated with the calcium booster. In this case, a mineralized layer of around 4 to 5 µm can be observed in this micrograph.

Table 2 displays the elemental mapping as a function of the treatment of dental tissues with the association between fluoride–silicon-rich toothpaste and the calcium booster. EDS detected an increase in the calcium signals after immediate treatment (7.0 weight%) when compared to control, untreated dentin (4.1 weight%). Similar results were observed for the phosphate signals as functions of the different evaluation points (control: 2.2 weight%; immediate: 3.7 weight%; and after 5 days: 1.7 weight%). Stronger silicon signals were detected at the surface areas of dentin after immediate treatment (5.5 weight%), which remained at a similar percentage at the 5-day evaluation (5.8 weight%). Conversely, the calcium signals after the 5-day evaluation were similar to those detected in the control, untreated dentin (4.3 and 4.1 weight%, respectively). Small amounts of potassium and sodium were only detected at the immediate evaluation (0.2 and 0.1 weight%).

Silicon signals were also detected at the surface areas of enamel after immediate treatment (0.6 weight%), which increased at the 5-day evaluation (2.2 weight%). In spite of a decrease in the weight% at the 5-day evaluation, the equivalent proportions of calcium and phosphate signals were detected when the results of both evaluation times were compared. No signal of carbon was detected in the untreated control enamel. Conversely, strong signals were detected after the immediate evaluation and after the 5-day evaluation (33.6 and 18.7 weight%, respectively). A similar comparison was observed for potassium, although at a weaker magnitude (0.3 and 0.6 weight%).

## 4. Discussion

The data presented here are from a complementary study that aimed to evaluate the mechanism of action of a fluoride–silicon-rich toothpaste containing REFIX technology, now associated with a calcium booster. It consists of a multifunctional phosphate-based dental gel that is stabilized in an acidified phosphate/fluoride combination that is created in the saliva [24]. This mixture enables the creation of new calcium/phosphate/fluorine-containing minerals, enhancing the enamel surface and remineralizing the subsurface carious lesion [22]. Due to the proposed technology, this product has an acidic pH, which causes calcium phosphate crystals to form in the acidic environment [24,25]. Previous in vitro and clinical studies have demonstrated the effectiveness of this toothpaste, either through its ability to remineralize the dental tissues or by clinically reducing the dentin hypersensitivity by occluding the dentinal tubules [11,25,26,27]. 

In spite of the alleged effectiveness of the REFIX technology, reasons that explain the analyses performed in the present in vitro study rely on the need for more information, as this association between the fluoride–silicon-rich toothpaste and calcium booster is indicated for chair-side professional application, aiming to obtain an immediate clinical effect and accelerate the formation of a mineralized layer onto the dental tissues (manufacturer’s information). Calcium boosters use the technology of calcium nanoparticles associated with phosphate salts [20], as demonstrated in Table 1. It has also been reported that boosters can supplement or represent an alternative to fluoride, considered a cost-effective strategy for caries prevention [28]. Furthermore, it is expected that boosters would ideally enhance the efficacy of fluoride at a lower concentration, decrease the risks of fluorosis, and also allow access to high-caries-risk populations [16]. Still, since calcium boosters reinforce the tooth structure by increasing the size of hydroxyapatite crystals, an understanding of the mechanism by which a biomimetic/bionic enamel-like structure is formed is extremely important, especially in hypomineralized teeth [29]. However, the biomimetic action of this association has not fully been established.

In a previous in vitro study [12], a booster containing calcium silicate salts and sodium phosphate associated with sodium fluoride was developed in order to improve the mechanism of action of toothpaste in the treatment of early erosive lesions. The authors speculated that the silicon-rich layer would not only promote the remineralization of eroded enamel but also act as a protective layer on the enamel surface. However, although a favorable effect was observed in dentin microhardness, this association was not able to protect against repetitive erosive and abrasive challenges. Up to now, no research studies are available on the topic regarding the technology related to the association of fluoride–silicon-rich toothpaste with the calcium booster, which is based on the content of calcium carbonate and tricalcium phosphate (manufacturer’s information).

Considering the dentin hypersensitivity, the association between fluoride–silicon-rich toothpaste/calcium booster also represents a viable alternative for periodontal health after basic and maintenance periodontal therapy [27]. Based on the previous explanation, the purpose of professional application in periodontal patients is to speed up the remineralization of the dentin tissue by depositing a mineral layer, gradually occluding the dentinal tubules. This relationship is expected to enable the faster deposition of phosphate and calcium ions at the surface of exposed tubules to prevent dentinal hypersensitivity, as demonstrated with the use of toothpaste alone [25], providing a more effective treatment for some indications.

In the present study, treating the dentin with toothpaste/calcium booster promoted the formation of a mineralized area immediately after brushing, exhibiting partially open dentin tubules (Figure 1A) in comparison to the untreated, control dentin (Figure 1B). Similar results were observed for the treated enamel (Figure 1C) compared to the untreated specimens (Figure 1D). An interesting result was observed in Figure 2, demonstrating a mineralized layer formed onto both the enamel and dentin surfaces after five daily applications of toothpaste/calcium booster. The mineralized layer that formed was around 4 to 5 µm for enamel and circa 7 µm for dentin. It was also demonstrated that there were mineralized areas inside the tubules, partially filling some of these tubules. Previous studies, evaluating the surface morphology of the dental tissues treated with the toothpaste containing REFIX alone, demonstrated a mineral layer onto the treated enamel surface of ~14 µm [30], and of 3 µm in dentin after a 7-day brushing evaluation [11].

Boosters of fluoride or an alternative to fluoride are incorporated into different delivery vehicles, such as toothpastes, and are intended to increase the concentration of calcium and phosphate delivered to caries lesions and/or increase their concentrations in plaque and saliva [16]. At an acidic pH, the benefit of delivering extra calcium provided by calcium boosters seems to improve the saturation of calcium in both plaque and saliva with respect to the tooth. In this manner, by increasing the saturation of calcium, it may enhance the presence of fluoride at the tooth surface and promote fluoride-induced remineralization [16]. 

Table 2 demonstrated an increase in the calcium signals in dentin after immediate treatment (7.0 weight%) compared to the untreated control group (4.1 weight%). The same was observed for the phosphate signals. In addition, stronger silicon signals were detected in dentin after immediate treatment (5.5 weight%), which remained at a similar percentage at the 5-day evaluation (5.8 weight%). Given the organic phase in dentin (30 vol%, mainly type I collagen) [31], the combination of fluoride–silicon-rich toothpaste and calcium booster appears to allow interactions between the negatively charged surface of SiO^2^ (–SiOH^2+^, –SiOH, –SiO) and positively charge-carried collagen, which is pH-dependent [32]. Electrostatic interactions, hydrogen bonding, and complexation all play a role in these interactions [33]. Adsorption at the protein–silica interface appears to favor the formation of hybrid materials through the copolymerization of silica precursors and collagen monomers, with collagen acting as a substrate or organic scaffold [32]. In this manner, the interactions provided by the treatment of the dentin with the combination of silicon-rich toothpaste/calcium booster in an acidic medium seem to favor the “silicification” of the collagen fibrils, which functioned as an organic scaffold for dentin tubule occlusion in the presence of a calcium-saturated medium. It can be speculated that associating the calcium booster with brushing the teeth with the fluoride–silicon-rich toothpaste might provide relevant results in other in vitro and clinical studies, considering the previously proven positive action of REFIX technology to regenerate the dental tissues in previous in vitro studies [24,30], as well as the fast and efficient clinical minimization of the dentin hypersensitivity symptoms [25,26].

Results displayed in Table 2 also demonstrated silicon signals in enamel at the surface areas after immediate treatment (0.6 weight%), which increased at the 5-day evaluation (2.2 weight%). In a previous study [11], similar signals of silicon were detected after 1 week of treatment with the fluoride–silicon-rich toothpaste alone (0.56 weight%). Conversely, after a 5-day treatment with both toothpaste/calcium booster, the detected signal of silicon in the present study was almost four times stronger. 

One of the results of the elemental analysis that caught our attention was the strong signal of carbon after the immediate evaluation and after the 5-day evaluation (33.6 and 18.7 weight%, respectively). Conversely, no carbon signals were detected in the untreated control enamel. Numerous carbon groups are present in the crystals of hydroxyapatite as carbonates. Carbonate can either replace the OH-ions in the hydroxyapatite structure to generate type A carbonate apatite crystals or the PO4^3-^ions to form type B apatite crystals [34]. Carbonate ions can also replace prevalently occurring phosphate groups, producing a persistent coating of carbonate–hydroxyapatite deposition on the enamel surface [35]. Conversely, it has been reported that the remineralization of the enamel surface treated with fluoride is mainly based on chemical–physical modifications rather than allowing the formation of a new layer [35].

## 5. Conclusions

The results of the present study demonstrated layer deposition with just one application, obliterating tubules with an enamel-like structure promoted via the association between the fluoride–silicon-rich toothpaste and the calcium booster. In this manner, it appears to be a promising strategy in the clinical management of mineral loss, caries, erosion, tooth sensitivity, and molar incisor hypomineralization (MIH). In vitro, in situ, and clinical studies are required to corroborate the results of the present study. Despite the limitations of the current study, it could be an alternative to using bovine teeth, although extracted human teeth could produce more relevant results. Additionally, other products, such as a gold-standard toothpaste or the REFIX-technology toothpaste alone, would enable additional useful comparisons. Although tubule occlusion was evident, it is crucial to assess the long-term effects of tubule occlusion to determine whether they would withstand repeated acid challenges and saliva immersion. Additional research is still required to assess the regenerative and protective effects on dental tissues of a fluoride–silicon-rich toothpaste combined with a calcium booster, particularly in in situ and in vivo studies.

## Figures and Tables

**Table 1:** Composition of the toothpastes selected for the study *.

Dentifrice	Composition	pH **
Regenerador Sentitive	1450 ppm sodium fluoride, glycerin, silica, sorbitol, sodium lauryl sulfate, aqua, aroma, PEF-12, cellulose gum, phosphoric acid, xylitol, tetrasodium pyrophosphate, sodium saccharin, triclosan, menthol, mica, sodium benzoate, REFIX Technology.	4.7
Calcium Booster	Dentifrice containing 5% calcium mix (calcium carbonate, tricalcium phosphate), silica, glycerin, CPC, saccharine, water).	7.8

* manufacturer: DentalClean, Londrina, PR, Brazil. ** manufacturer’s information.

**Table 2:** Mean percent values of elemental mapping (W = weight; A = atomic).

	Dentin						Enamel					
	Untreated		Treated Immediate		Treated after 5 Days		Untreated		Treated Immediate		Treated after 5 Days	
Element *	W%	A%	W%	A%	W%	A%	W%	A%	W%	A%	W%	A%
K L	-	-	0.2	0.1	-	-	-	-	0.3	0.2	0.6	0.3
C K	49.2	75.4	31.6	52.0	32.3	48.5	-	-	33.6	53.7	18.7	32.9
Ca L	4.1	1.9	7.0	3.5	4.3	1.9	33.6	21.0	13.8	6.6	12.3	6.4
O K	16.3	18.8	28.9	35.8	38.4	43.2	40.4	63.3	27.5	32.9	39.7	52.2
Na K	-	-	0.1	0.1	-	-	0.4	0.5	0.5	0.4	1.1	1.0
Si K	-	-	5.5	3.9	5.8	3.7	-	-	0.6	0.4	2.2	1.6
P K	2.2	1.3	3.7	2.4	1.7	1.0	17.2	13.9	6.3	3.9	5.9	4.0

* K—potassium; C—carbon; Ca—calcium; O—oxygen; Na—sodium; Si—silicon; P—phosphorus.

## Data Availability

The data presented in this study are contained within the article.
